# Supplementary material for: Computational Design and Development of Benzodioxane-Benzamides as Potent Inhibitors of FtsZ by Exploring the Hydrophobic Subpocket
Source: Antibiotics (Basel). 2021 Apr 15;10(4):442. doi: 10.3390/antibiotics10040442 (PMC8071314; doi:10.3390/antibiotics10040442)
Supplement: Supplementary file 1 [file antibiotics-10-00442-s001.zip › antibiotics-1162609-supplementary.pdf]

Supporting Information

Computational Design and Development of Benzodioxane-Benzamides as Potent Inhibitors of FtsZ by Exploring the Hydrophobic Subpocket.

Valentina Straniero*§, Victor Sebastián-Pérez §, Lorenzo Suigo, William Margolin, Andrea Casiraghi, Martina Hrast, Carlo Zanotto, Irena Zdovc, Antonia Radaelli and Ermanno Valoti

Summary

3-[2-(7-(5-methyl-1,2,4-oxadiazol-3-yl)-1,4-benzodioxan-2-yl)ethyloxy]-2,6-difluorobenzamide (1) 3

Figure S1: ^{1}H-NMR spectrum of Compound 1 3

Figure S2: ^{13}C-NMR spectrum of Compound 1 3

Figure S3: HPLC chromatogram of Compound 1 4

3-[2-(7-(5-ethyl-1,2,4-oxadiazol-3-yl)-1,4-benzodioxan-2-yl)ethyloxy-2,6-difluorobenzamide (2) 5

Figure S4: ^{1}H-NMR spectrum of Compound 2 5

Figure S5: ^{13}C-NMR spectrum of Compound 2 5

Figure S6: HPLC chromatogram of Compound 2 6

3-[2-(7-(5-methylthio-1,2,4-oxadiazol-3-yl)-1,4-benzodioxan-2-yl)ethyloxy]-2,6-difluorobenzamide (3) 7

Figure S7: ^{1}H-NMR spectrum of Compound 3 7

Figure S8: ^{13}C-NMR spectrum of Compound 3 7

Figure S9: HPLC chromatogram of Compound 3 8

3-[(2,3-dihydronaphtho[2,3-b][1,4]dioxin-2-yl)methoxy]-2,6-difluorobenzamide (4) 9

Figure S10: ^{1}H-NMR spectrum of Compound 4 9

Figure S11: ^{13}C-NMR spectrum of Compound 4 9

Figure S12: HPLC chromatogram of Compound 4 10

3-[2-(2,3-dihydronaphtho[2,3-b][1,4]dioxin-2-yl)ethoxy]-2,6-difluorobenzamide (5) 11

Figure S13: ^{1}H-NMR spectrum of Compound 5 11

Figure S14: ^{13}C-NMR spectrum of Compound 5 11

Figure S15: HPLC chromatogram of Compound 5 12

3-[(3-(naphthalen-2-yl)propyl-1-oxy]-2,6-difluorobenzamide (6) 13

Figure S16: ^{1}H-NMR spectrum of Compound 6 13

Figure S17: ^{13}C-NMR spectrum of Compound 6 13

Figure S18: HPLC chromatogram of Compound 6 14

3-(2,3,6,7,8,9-hexahydronaphtho[2,3-*b*][1,4]dioxin-2-yl)methoxy]-2,6-difluorobenzamide (7) 15

Figure S19: ^{1}H-NMR spectrum of Compound 7 15

Figure S20: ^{13}C-NMR spectrum of Compound 7 15

Figure S21: HPLC chromatogram of Compound 7 16

3-[2-(2,3,6,7,8,9-hexahydronaphtho[2,3-*b*][1,4]dioxin-2-yl)ethoxy]-2,6-difluorobenzamide (8) 17

Figure S22: ^{1}H-NMR spectrum of Compound 8 17
Figure S23: ^{13}C-NMR spectrum of Compound 8 17
Figure S24: HPLC chromatogram of Compound 8 18

3-[2-(7-(5-methyl-1,2,4-oxadiazol-3-yl)-1,4-benzodioxan-2-yl)ethyloxy]-2,6-difluorobenzamide (1)

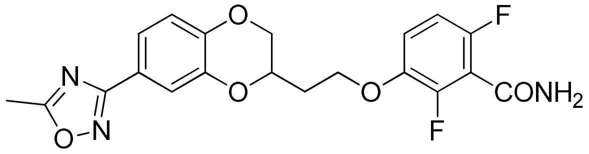

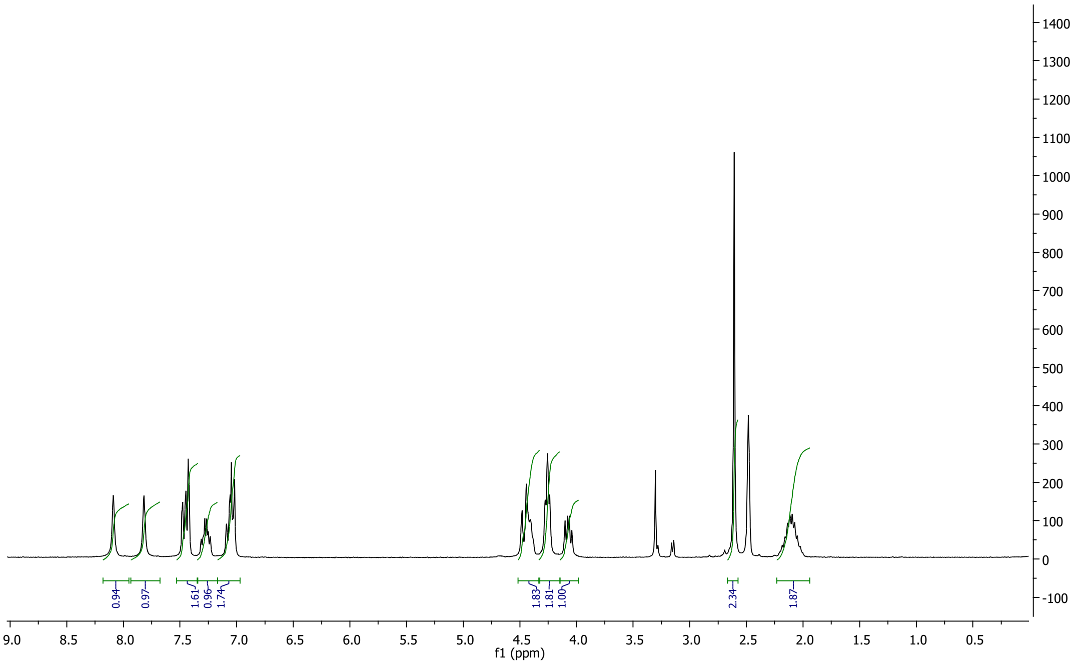

Figure S1: ¹H-NMR spectrum of Compound 1

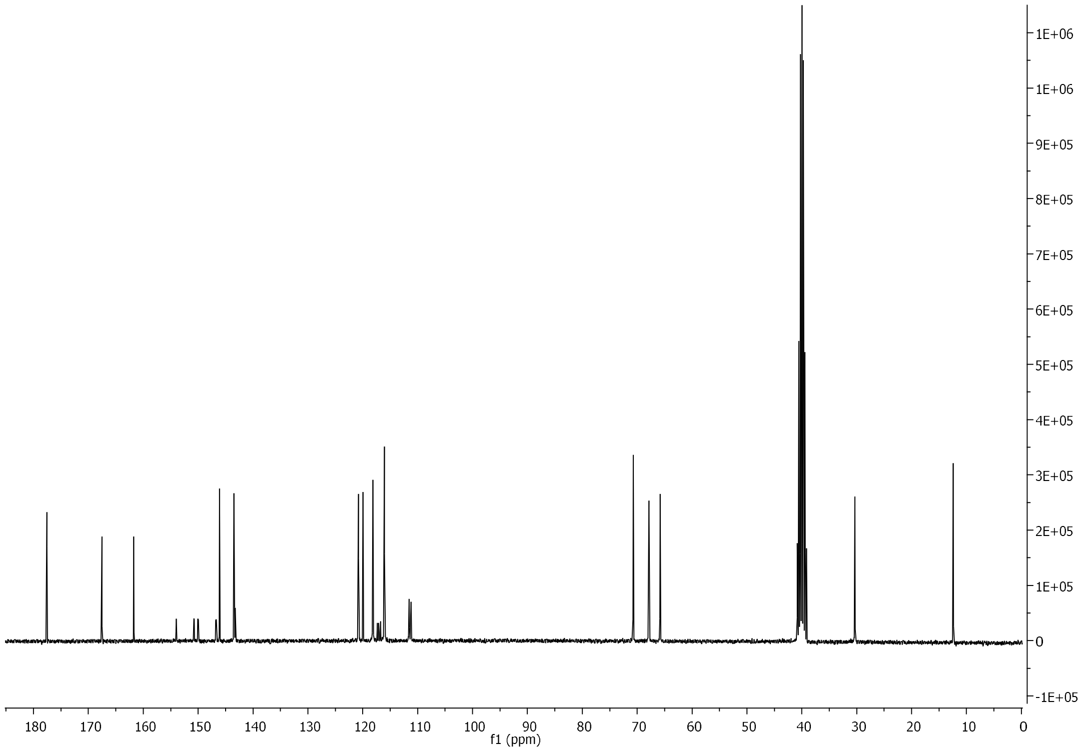

Figure S2: ¹³C-NMR spectrum of Compound 1

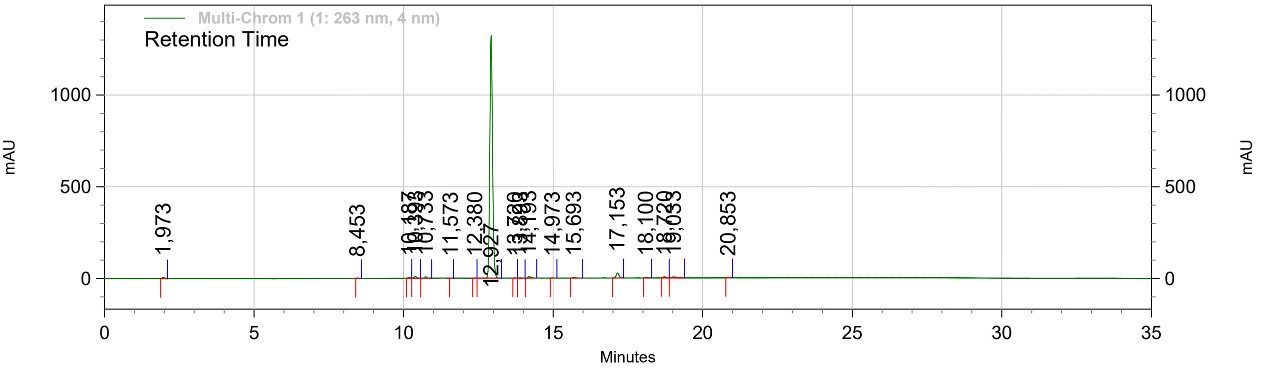

Figure S3: HPLC chromatogram of Compound 1

3-[2-(7-(5-ethyl-1,2,4-oxadiazol-3-yl)-1,4-benzodioxan-2-yl)ethyloxy-2,6-difluorobenzamide (2)

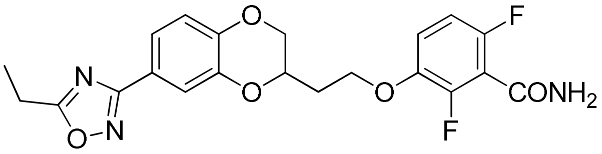

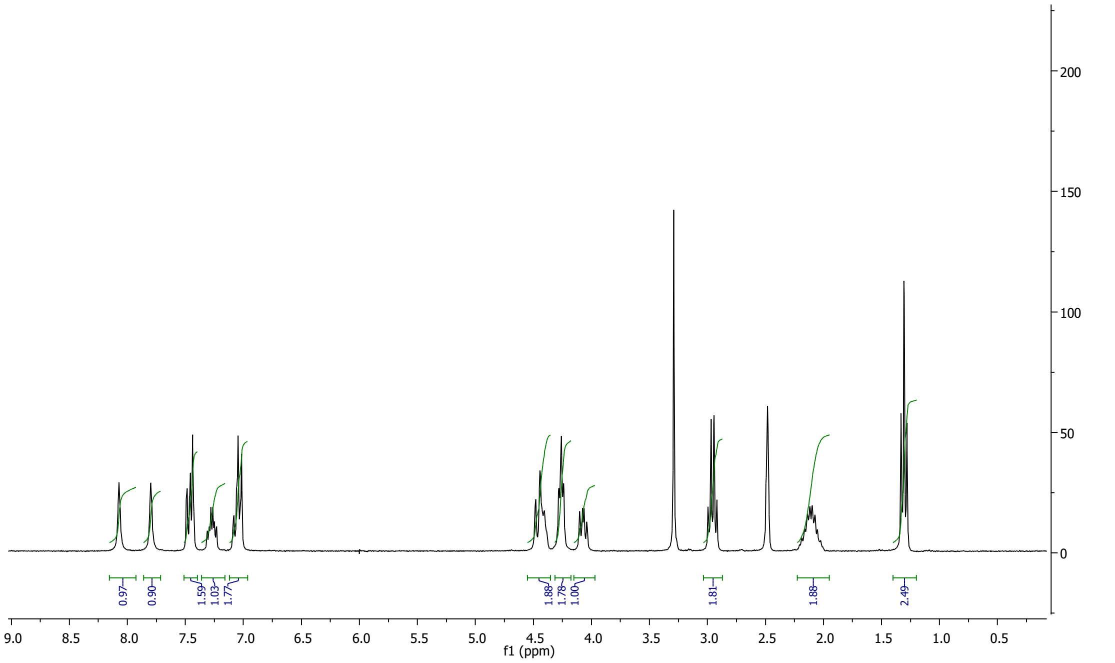

Figure S4: ^{1}H-NMR spectrum of Compound 2

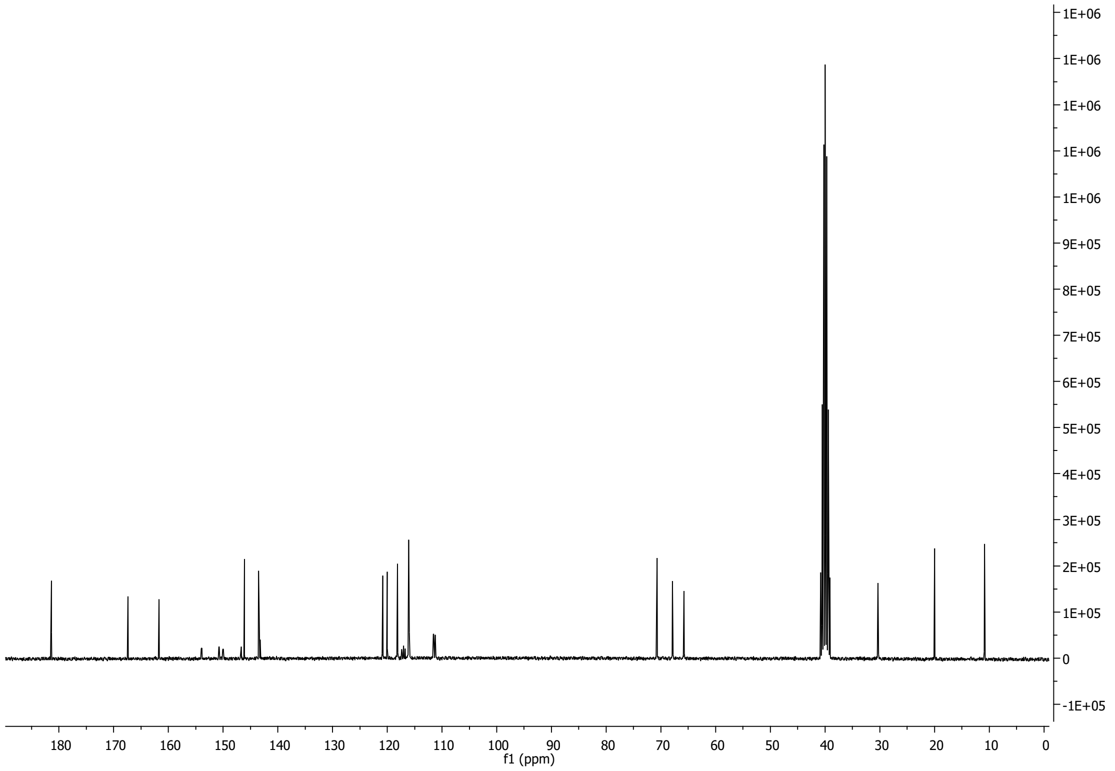

Figure S5: ^{13}C-NMR spectrum of Compound 2

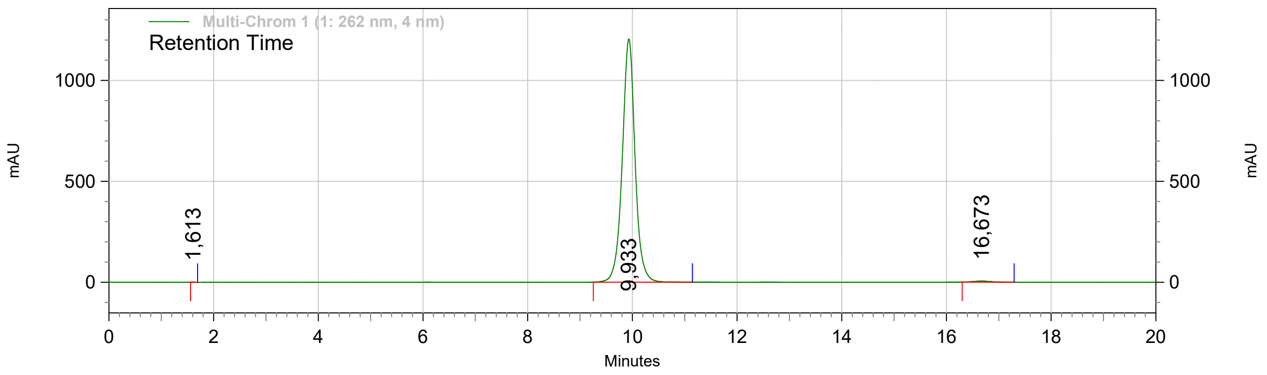

Figure S6: HPLC chromatogram of Compound 2

3-[2-(7-(5-methylthio-1,2,4-oxadiazol-3-yl)-1,4-benzodioxan-2-yl)ethyloxy]-2,6-difluorobenzamide (3)

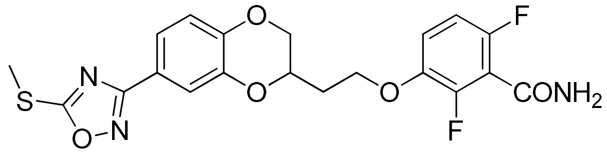

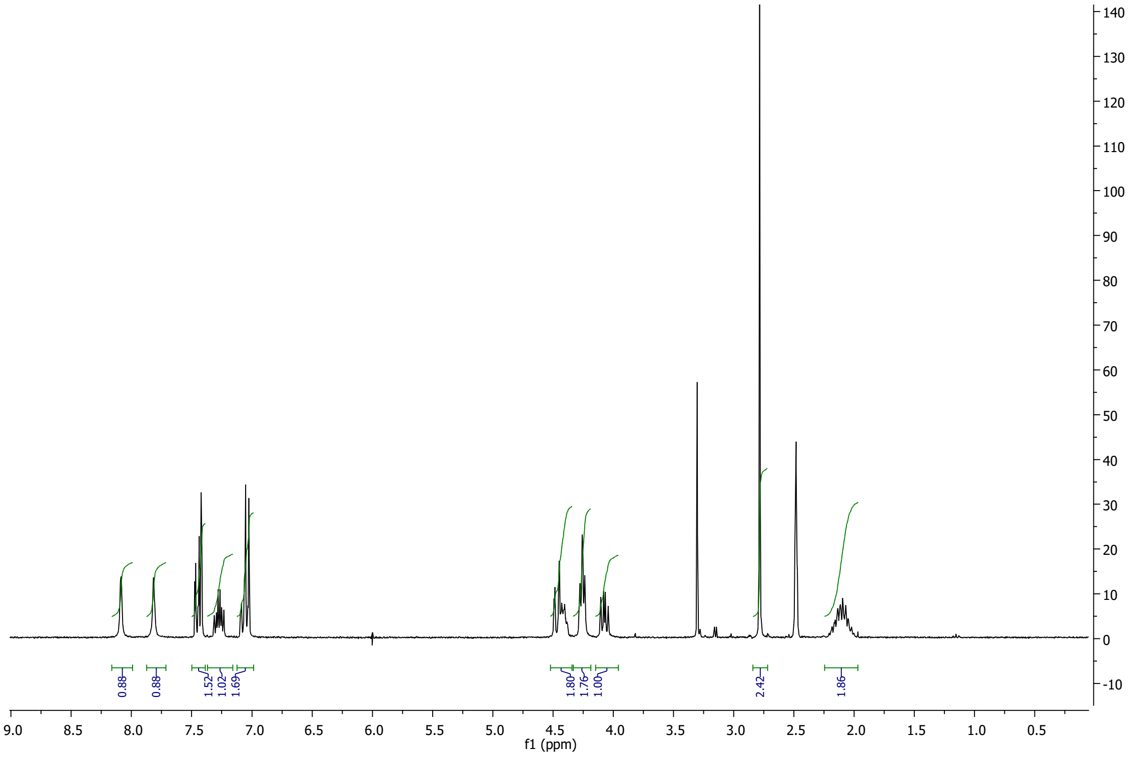

Figure S7: ^{1}H-NMR spectrum of Compound 3

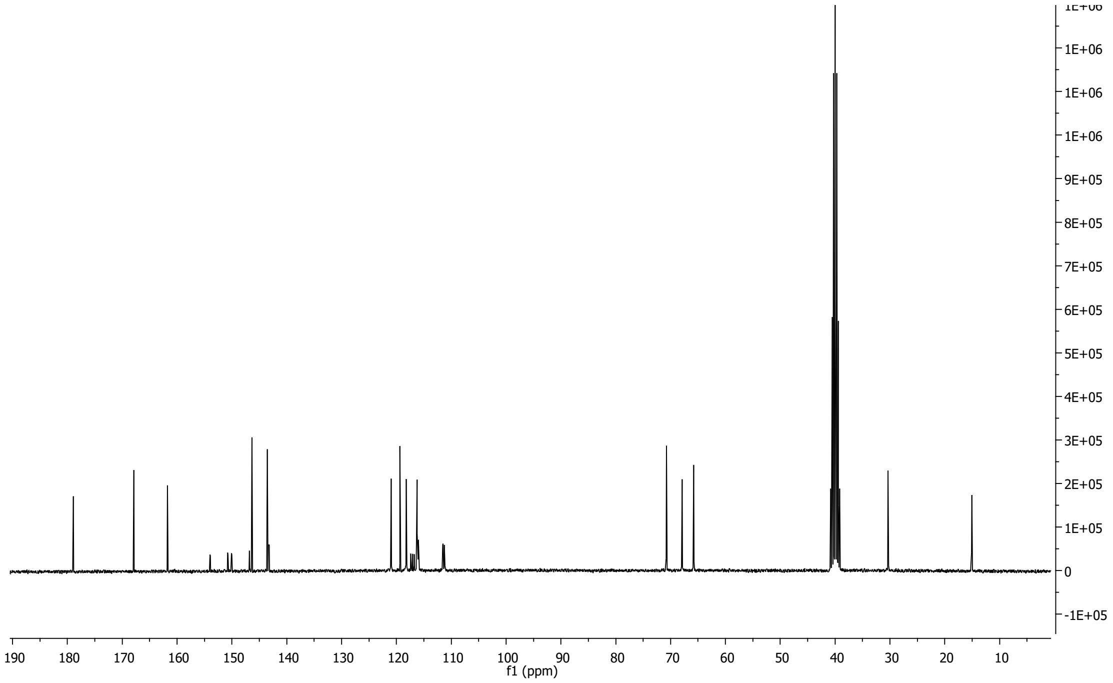

Figure S8: ^{13}C-NMR spectrum of Compound 3

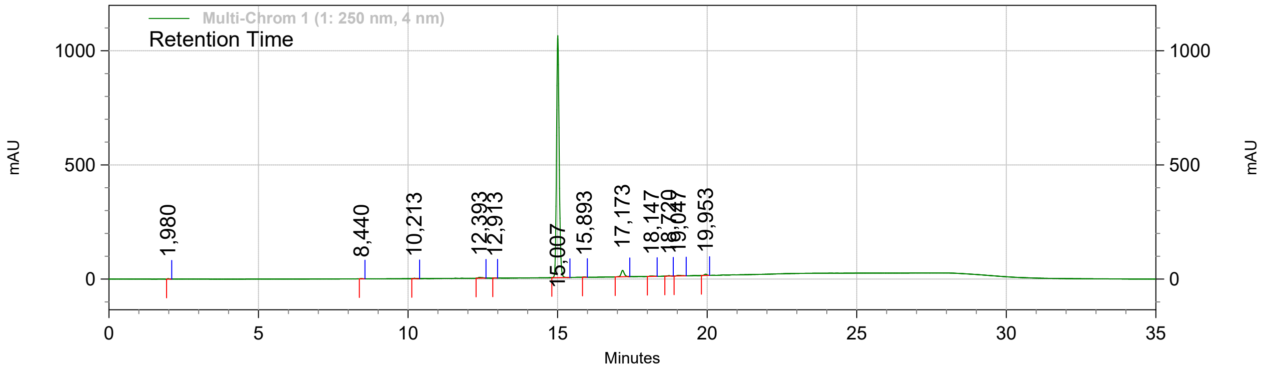

Figure S9: HPLC chromatogram of Compound 3

3-[(2,3-dihydronaphtho[2,3-b][1,4]dioxin-2-yl)methoxy]-2,6-difluorobenzamide (4)

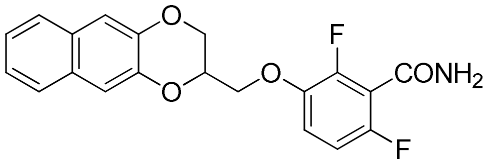

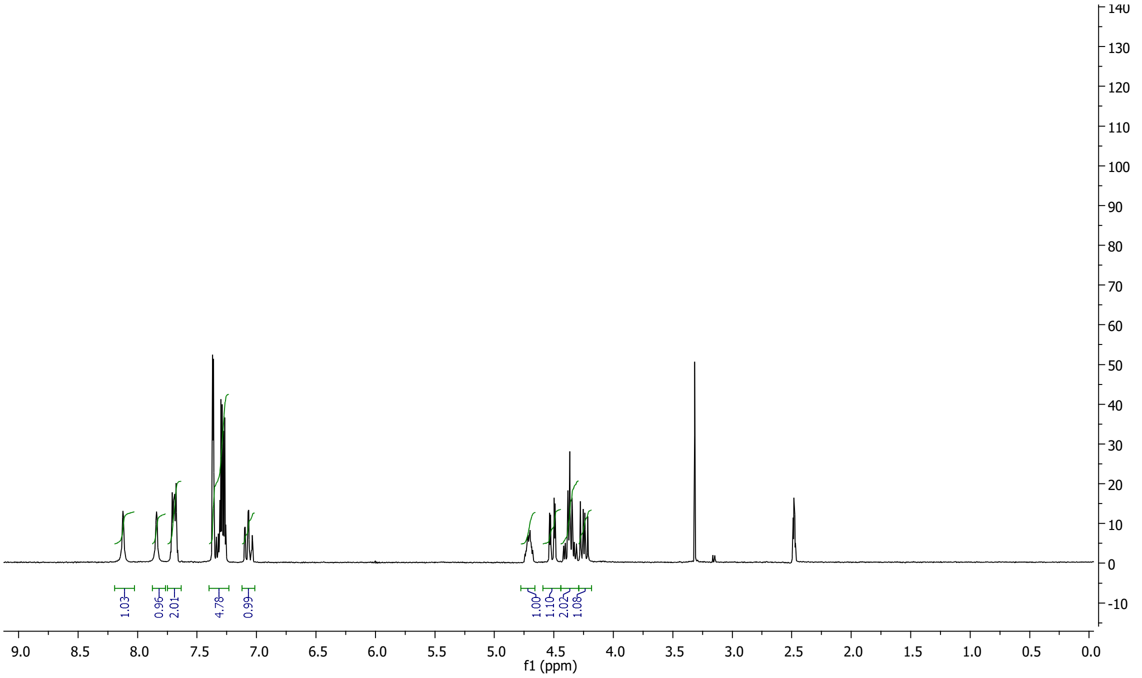

Figure S10: ¹H-NMR spectrum of Compound 4

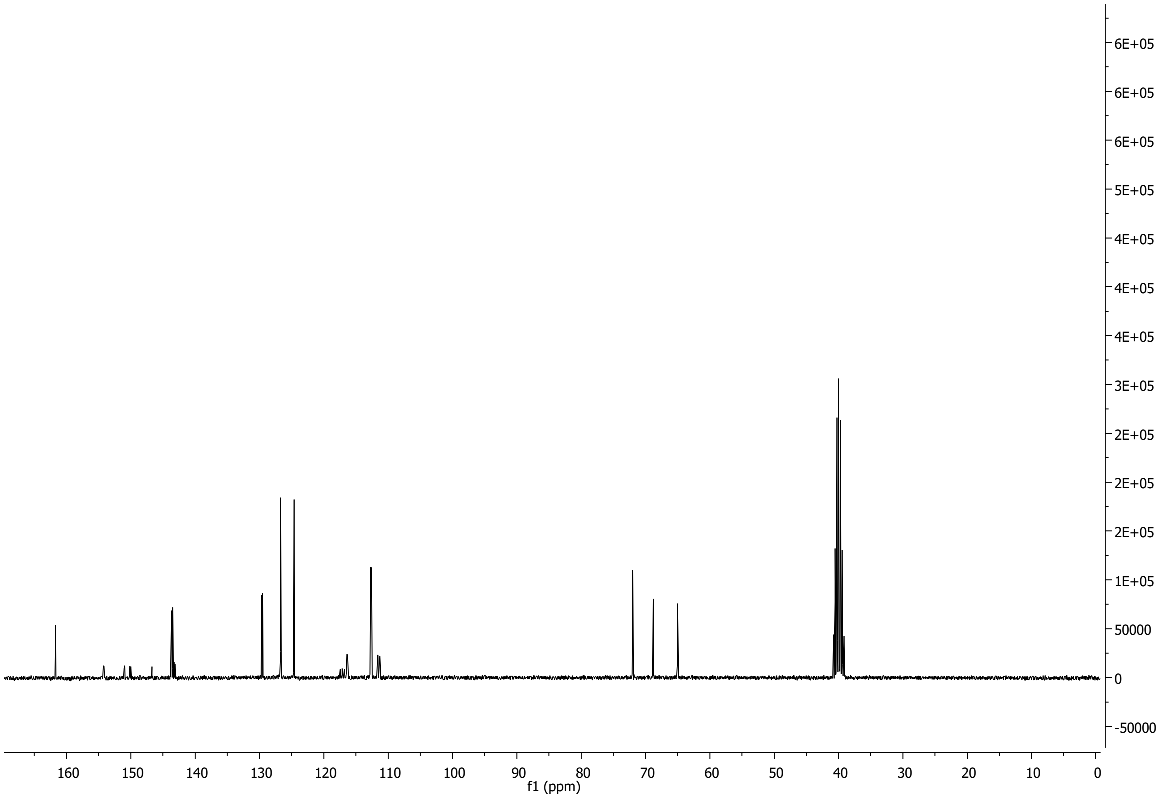

Figure S11: ¹³C-NMR spectrum of Compound 4

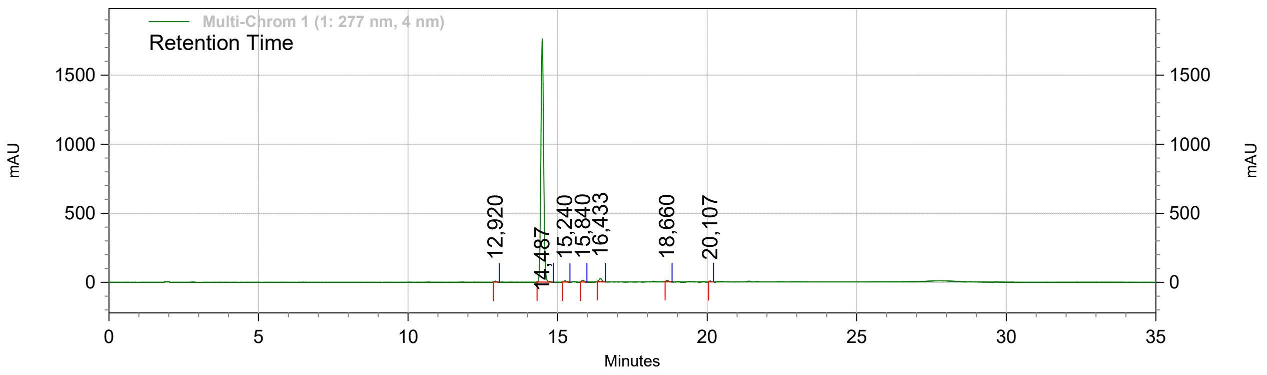

Figure S12: HPLC chromatogram of Compound 4

3-[2-(2,3-dihydronaphtho[2,3-b][1,4]dioxin-2-yl)ethoxy]-2,6-difluorobenzamide (5)

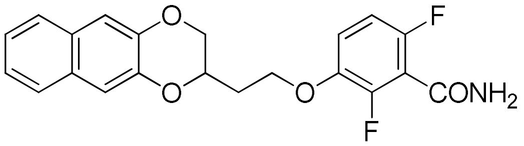

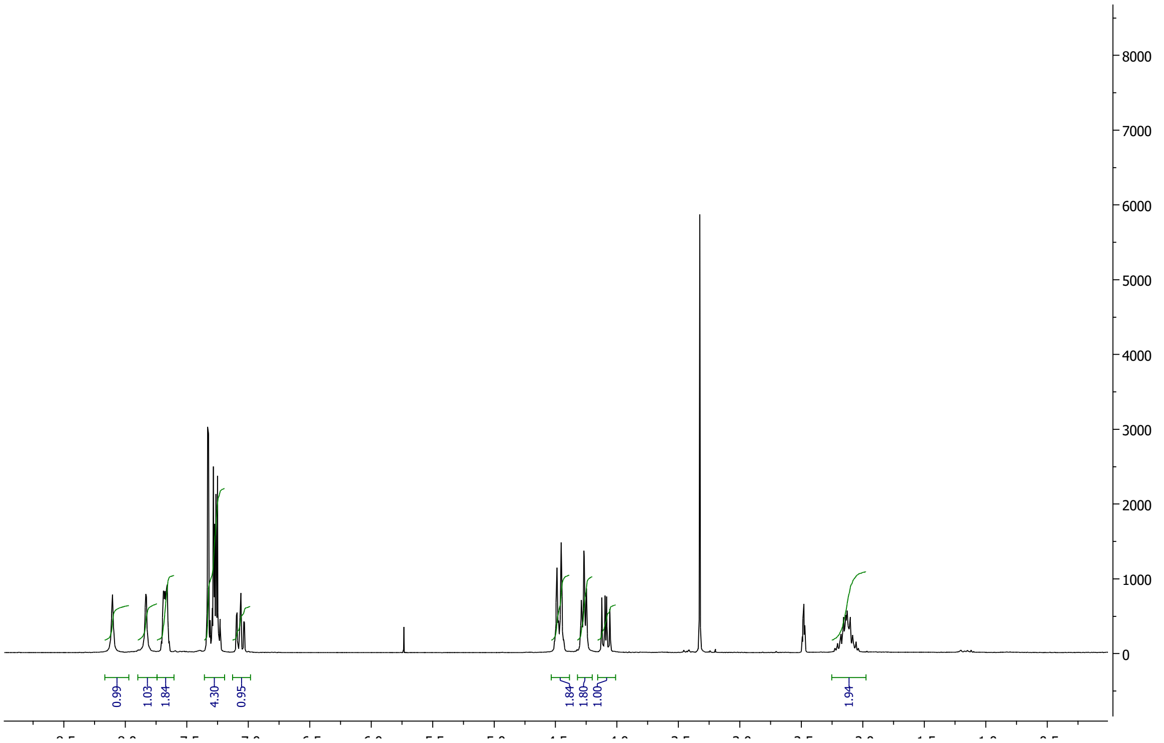

Figure S13: ^{1}H-NMR spectrum of Compound 5

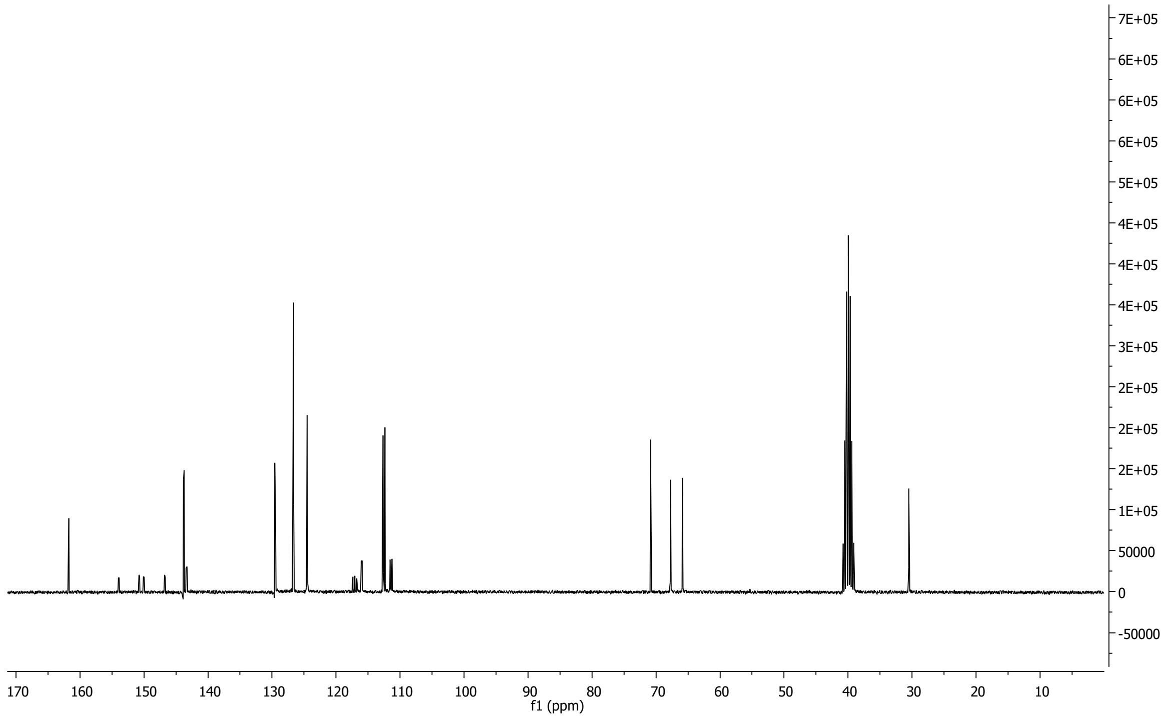

Figure S14: ^{13}C-NMR spectrum of Compound 5

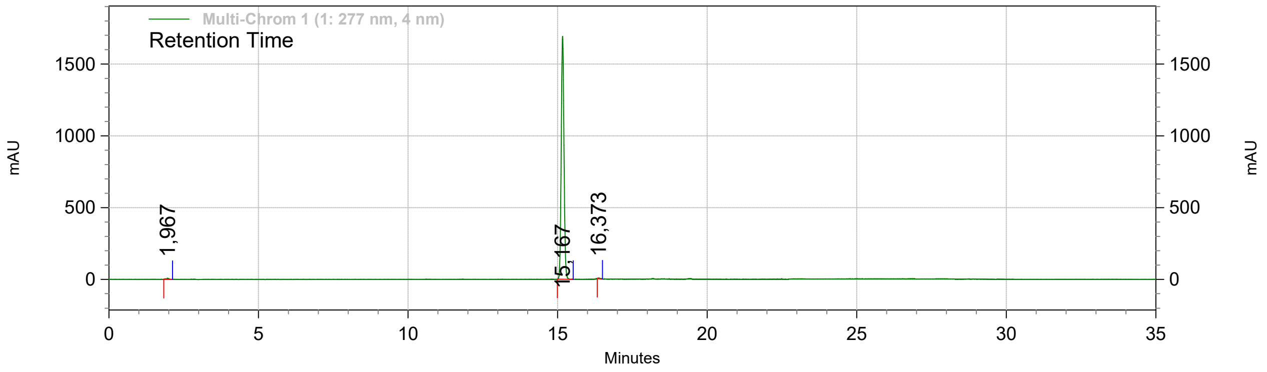

Figure S15: HPLC chromatogram of Compound 5

3-[(3-(naphthalen-2-yl)propyl-1-oxy]-2,6-difluorobenzamide (6)

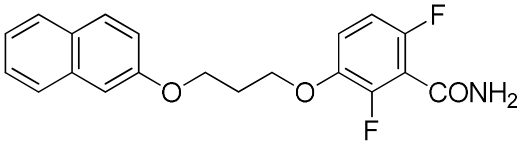

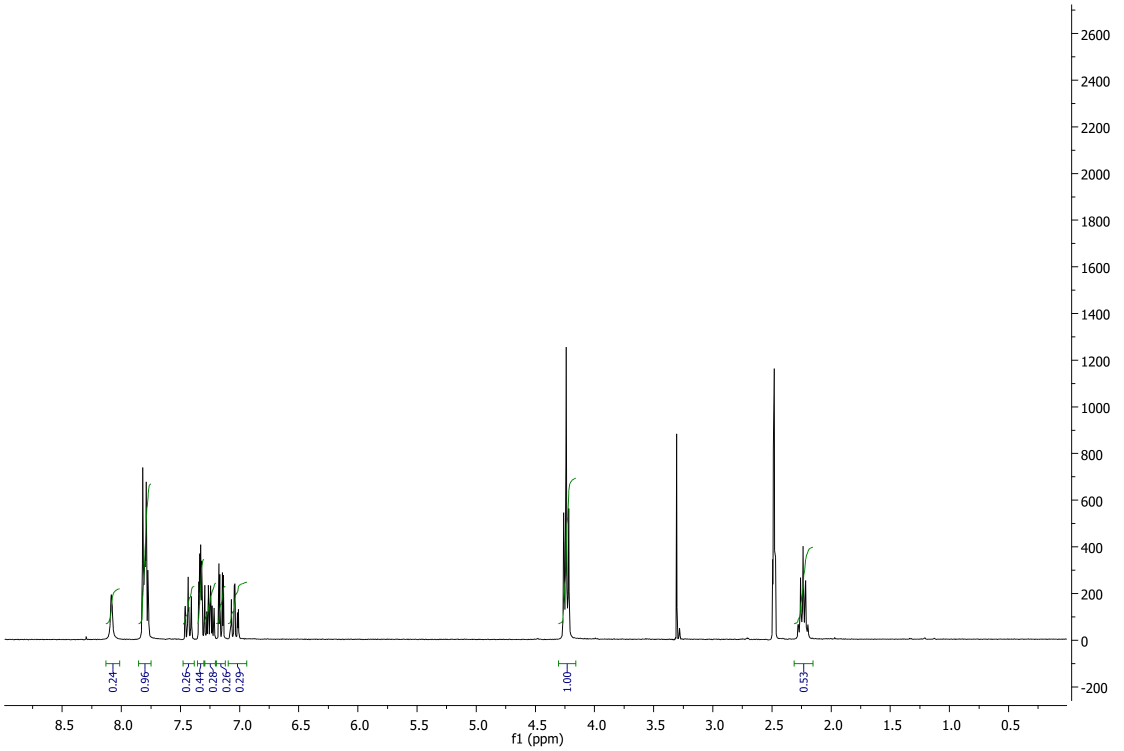

Figure S16: 1H-NMR spectrum of Compound 6

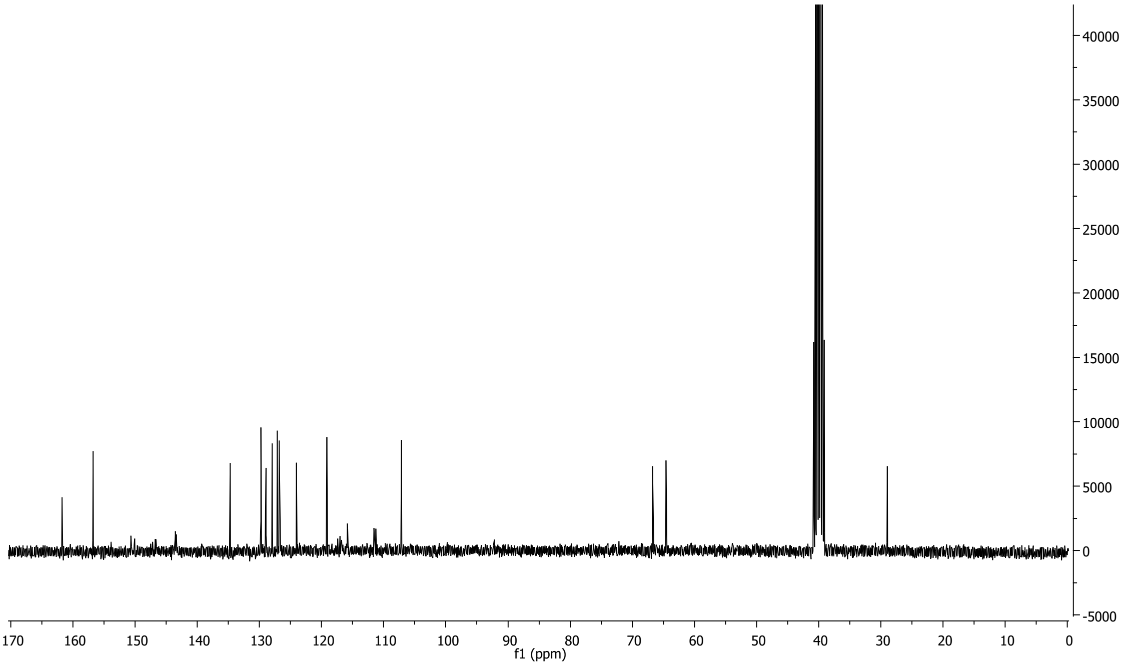

Figure S17: 13C-NMR spectrum of Compound 6

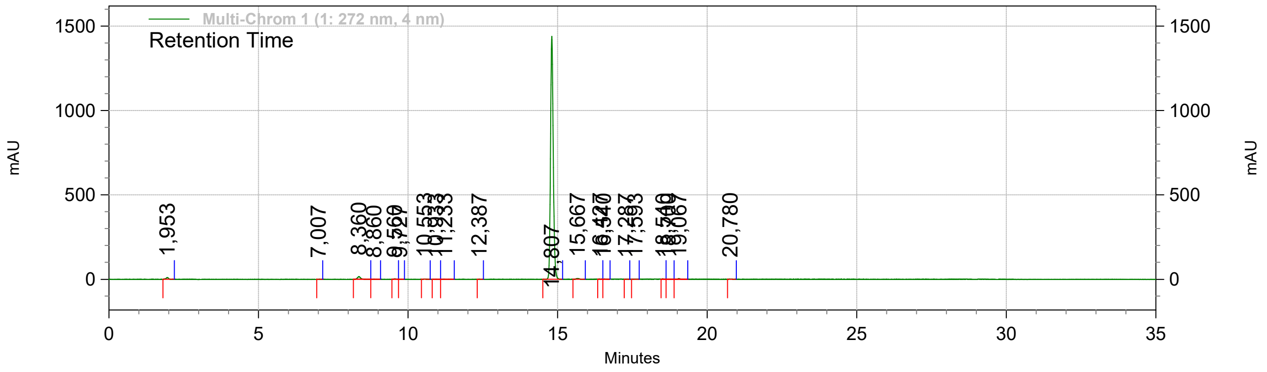

Figure S18: HPLC chromatogram of Compound 6

3-(2,3,6,7,8,9-hexahydronaphtho[2,3-*b*][1,4]dioxin-2-yl)methoxy]-2,6-difluorobenzamide (7)

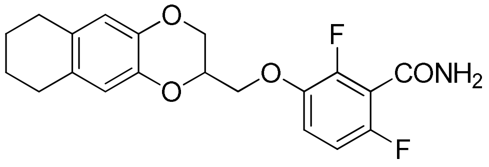

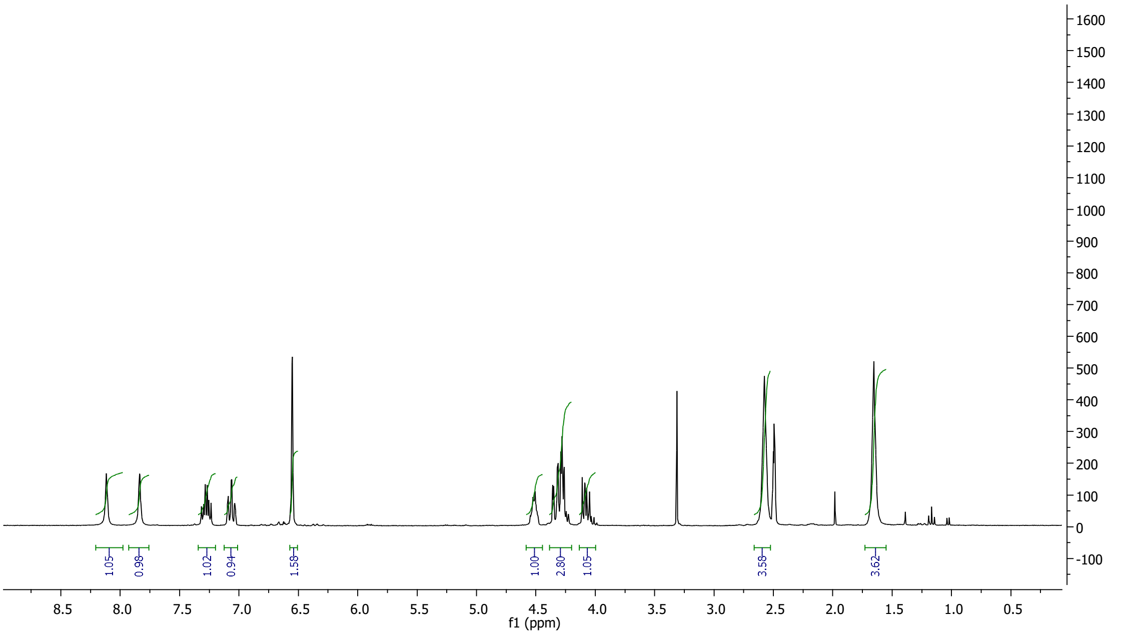

Figure S19: ^{1}H-NMR spectrum of Compound 7

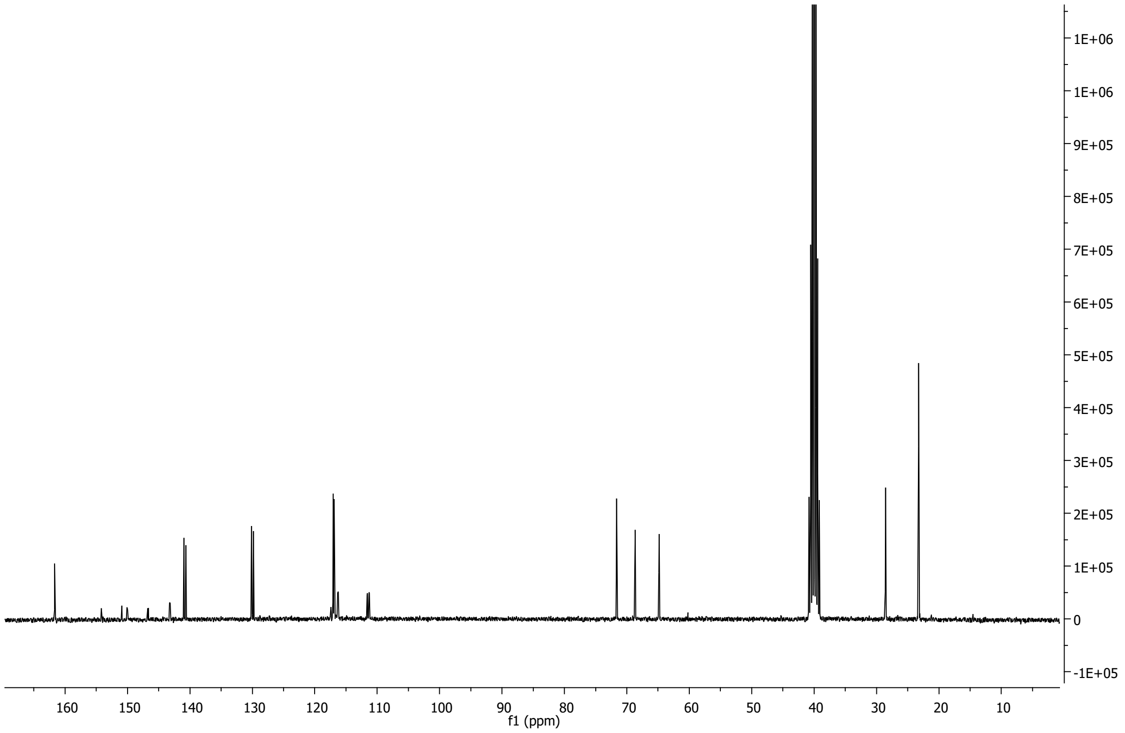

Figure S20: ^{13}C-NMR spectrum of Compound 7

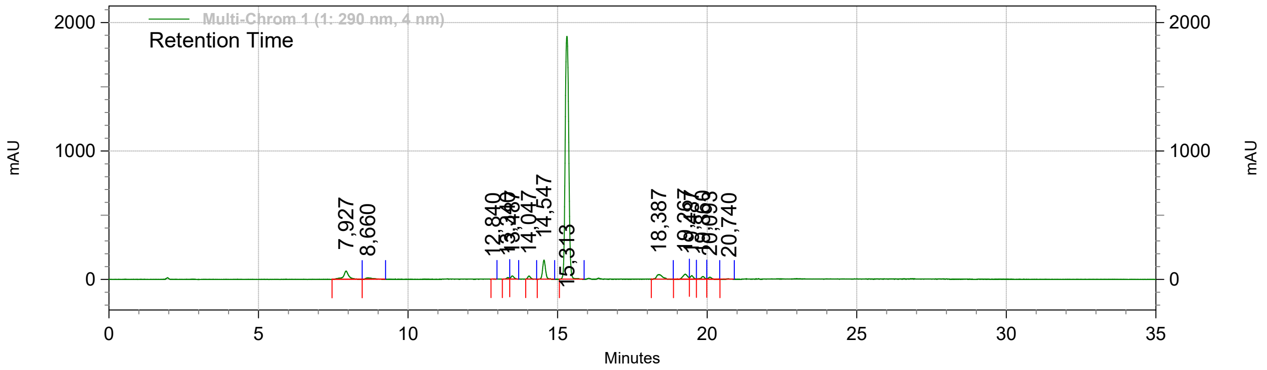

Figure S21: HPLC chromatogram of Compound 7

3-[2-(2,3,6,7,8,9-hexahydronaphtho[2,3-*b*][1,4]dioxin-2-yl)ethoxy]-2,6-difluorobenzamide (8)

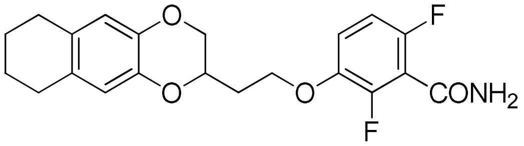

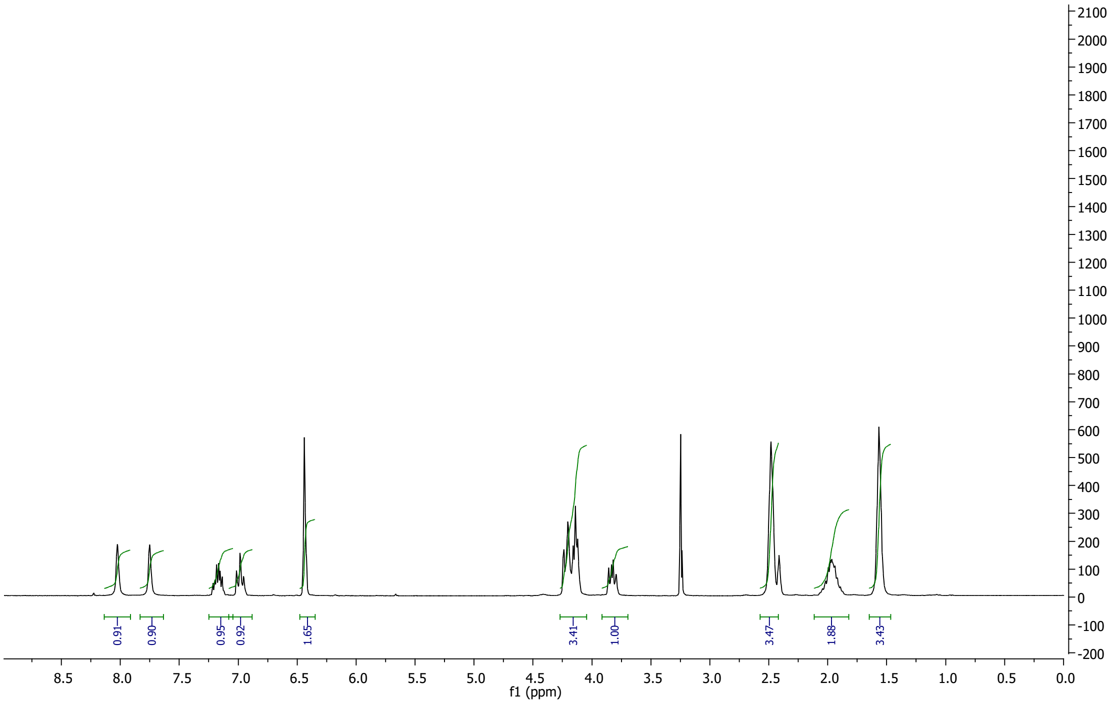

Figure S22: ^{1}H-NMR spectrum of Compound 8

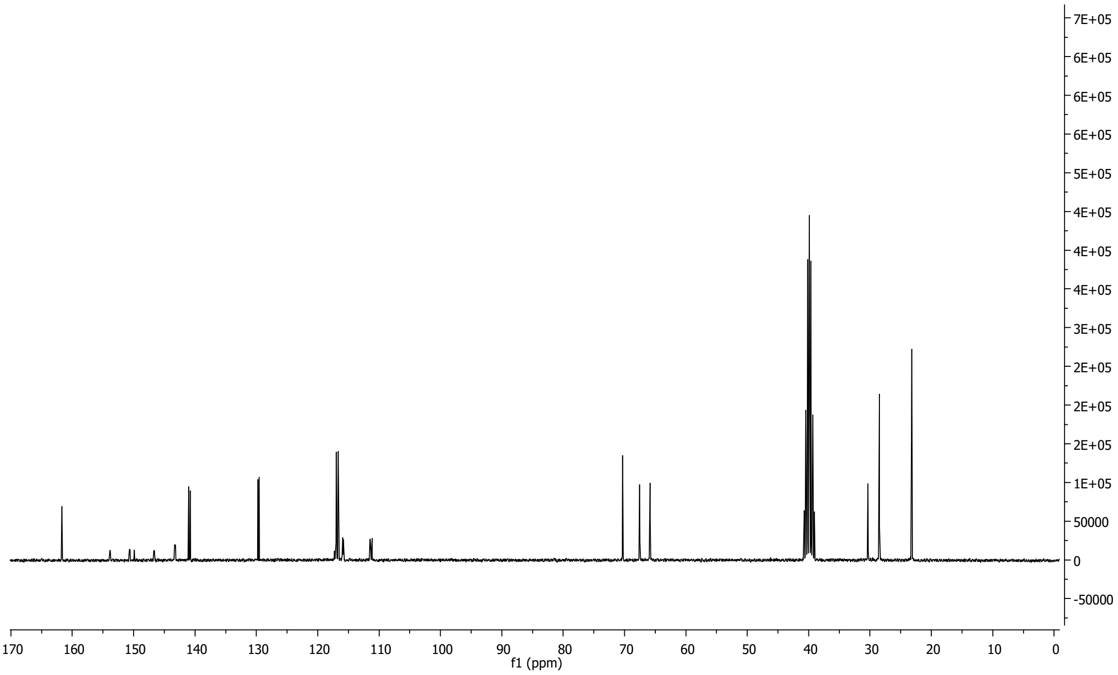

Figure S23: ^{13}C-NMR spectrum of Compound 8

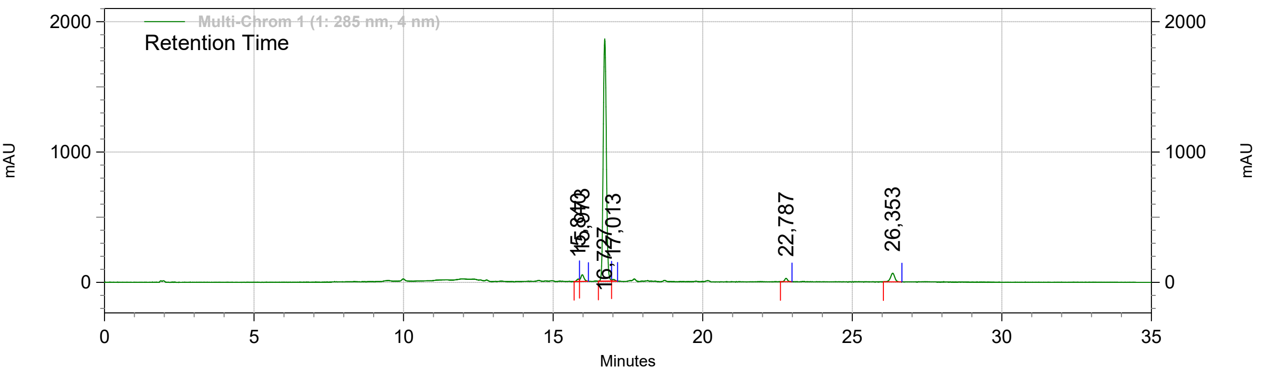

Figure S24: HPLC chromatogram of Compound 8
